# Genome sequence data of *Bacillus spizizenii* GM2: a lipopeptide producer

**DOI:** 10.1128/mra.01067-24

**Published:** 2025-01-13

**Authors:** Guzel Lutfullina, Daria Pudova, Marat Lutfullin, Anastasia Nikolaeva, Adela Abubakirova, Yaw Akosah, Elena Shagimardanova, Margarita Sharipova, Ayslu Mardanova

**Affiliations:** 1 Department of Microbiology, Institute of Fundamental Medicine and Biology, Kazan (Volga Region) Federal University, Kazan, Russia; 2 Laboratory of Agrobioengineering, Institute of Fundamental Medicine and Biology, Kazan (Volga Region) Federal University 5894 , Kazan, Russia; 3 Department of Molecular Pathobiology, New York University College of Dentistry, New York, USA; 4 Loginov Moscow Clinical Scientific Center, Moscow, Russia; SUNY College of Environmental Science and Forestry, Syracuse, New York, USA

**Keywords:** probiotics, genome sequencing, antimicrobial peptide, phylogenetic analysis, *Bacillus spizizenii*

## Abstract

We present the findings from the genome-sequencing project of *Bacillus spizizenii* GM2, sourced from rhizospheric soil and renowned for its lipopeptide production. The genome spans 4,216,713 base pairs with an average G + C content of 43,6%.

## ANNOUNCEMENT

The high antagonistic activity of *Bacillus* bacteria against various pathogens is attributed to their ability to synthesize a wide range of antimicrobial lipopeptides, such as surfactin, iturin, fengycin, polymyxin, and kurstakin (1). Their activity disrupts bacterial membranes, making it difficult for pathogens to develop resistance (2, 3). The described bacterium is a producer of antimicrobial lipopeptides, leading to the whole-genome sequencing of *Bacillus spizizenii* GM2 (4).

*B. spizizenii* GM2 was isolated from potato rhizosphere soil (GPS coordinates N55.615850°, E49.335570°). To isolate the bacterium, non-rhizosphere soil was removed from the roots by shaking. The roots (5 g) were placed in sterile phosphate-buffered saline (PBS) (50 mL) and shaken for 1 min to create a homogeneous suspension. Two milliliters of this suspension was centrifuged at 12,000 rpm for 2 min. A series of dilutions of the supernatant were prepared and plated on Luria Bertani Agar (LA) (10.0 g tryptone, 5.0 g yeast extract, 5.0 g NaCl, 20 g agar; 1,000 mL water at pH 6.8–7.2), with cultures incubated for 48 h at 37°C (5).

Genomic DNA from *B. spizizenii* strain GM2 was extracted using the NucleoSpin Microbial DNA Extraction Kit. DNA concentration was measured with a NanoDrop 2000c Spectrophotometer (Thermo Scientific), yielding 500 ng/µL. Whole-genome sequencing and analysis of genes related to antimicrobial peptide biosynthesis were performed. DNA fragmentation was conducted using Covaris S220. Library construction was carried out with the NEBNext Ultra II DNA Library Prep Kit (New England Biolabs). Quality assessment of DNA fragmentation and library preparation was performed using a 2100 Bioanalyzer (Agilent Technologies) and a high-sensitivity DNA kit (Agilent Technologies). DNA sequencing was executed on the Illumina MiSeq platform (Illumina) with a 301 bp paired-end library. The quality of sequencing data was evaluated using FastQC_v0.11.3 software. As a result, 12,860,230 raw reads were obtained. After filtration with Trimmomatic v.0.32 (parameters: HEADCROP:10, CROP:260, SLIDINGWINDOW:4:20, MINLEN:50), 11,820,346 reads were used for genome assembly. *De novo* assembly and analysis of contigs were performed using SPAdes_v3.8.1 with the “--careful” parameter. The genome assembly consisted of 24 contigs (≥500 bp) with a total length of 4,216,713 bp and a GC content of 43.6%. The N50 value was 447,231 bp, L50 was 3 bp, and coverage was 274×.

Average nucleotide identity (ANI) was calculated using JSpeciesWS (https://jspecies.ribohost.com/jspeciesws/). Comparative ANIm analysis of the *Bacillus* GM2 genome with 14 complete genomes from three *Bacillus* species revealed the closest relatedness to *B. spizizenii* (homology >95%). Annotation was added by the NCBI Prokaryotic Genome Annotation Pipeline (released 2013) (6). The genome was annotated using the NCBI Prokaryotic Genomes Annotation Pipeline, resulting in 4,313 annotated genes: 4,077 protein-coding genes, 82 tRNA genes, 28 rRNA genes, and 121 pseudogenes. Using the antiSMASH program version 7.1.0 (7), 11 gene clusters related to secondary metabolite synthesis were identified in the *B. spizizenii* GM2 genome (Table 1).

**TABLE 1:** List of putative antimicrobial peptides in *Bacillus spizizenii* GM2

Contig	Region	Type	From	To	Most similar known cluster
1	1.1	Trans-acyltransferase polyketide synthase (Trans-AT PKS) Other types of polyketide synthase (PKS) Type III PKS Non-ribosomal peptide synthetase	231,116	345,969	Bacillaene_biosynthetic_gene_cluster (100% of genes show similarity)
1	1.2	Non-ribosomal peptide synthetase Beta-lactone containing protease inhibitor Trans-AT PKS	439,950	517,178	Mycosubtilin_biosynthetic_gene_cluster (100% of genes show similarity)
1	1.3	Terpene	590,056	611,954	–^*a*^
1	1.4	Type III PKS	660,324	701,439	–
2	2.1	Trans-AT PKS	1	81,102	Macrolactin_biosynthetic gene cluster (100% of genes show similarity)
3	3.1	Cluster containing a secondary metabolite-related protein that does not fit into any other category	5,742	47,160	Bacilysin_biosynthetic_gene_cluster (100% of genes show similarity)
3	3.2	Sactipeptide	50,750	72,359	Subtilosin_A_biosynthetic_gene_cluster (100% of genes show similarity)
3	3.3	tRNA-dependent cyclodipeptide synthases	302,398	323,144	Pulcherriminic acid biosynthetic gene cluster (100% of genes show similarity)
4	4.1	Non-ribosomal peptide metallophores Non-ribosomal peptide synthetase	175,234	227,016	Bacillibactin_biosynthetic_gene_cluster (100% of genes show similarity)
5	5.1	Glycosylated lanthipeptide/linaridin hybrids like MT210103	54,221	96,105	
11	11.1	Non-ribosomal peptide synthetase	27,533	92,923	Surfactin_biosynthetic_gene_cluster (82% of genes show similarity)

^*a*^ "_" was used to depict the absence of a cluster.

In conclusion, this study undertook the genomic sequencing of a rhizospheric strain, *B. spizizenii* GM2, to unravel the genetic underpinnings of its probiotic potential.

## Data Availability

Data of whole genome shotgun sequencing project were uploaded to NCBI database under accession number NZ_NKAK00000000, BioProject accession number PRJNA391893 and BioSample accession number SAMN07277156. Accession number for the raw reads: SRR29665085.
